# Nucleosome footprinting in plasma cell-free DNA for the pre-surgical diagnosis of ovarian cancer

**DOI:** 10.1038/s41525-022-00300-5

**Published:** 2022-04-28

**Authors:** Adriaan Vanderstichele, Pieter Busschaert, Chiara Landolfo, Siel Olbrecht, An Coosemans, Wouter Froyman, Liselore Loverix, Nicole Concin, Elena Ioana Braicu, Pauline Wimberger, Els Van Nieuwenhuysen, Sileny N. Han, Toon Van Gorp, Tom Venken, Ruben Heremans, Patrick Neven, Tom Bourne, Ben Van Calster, Dirk Timmerman, Diether Lambrechts, Ignace Vergote

**Affiliations:** 1 Department of Obstetrics and Gynaecology, University Hospitals Leuven, Leuven Cancer Institute, Leuven, Belgium; 2 Department of Oncology, KU Leuven, Gynaecological Oncology, University Hospitals Leuven, Leuven, Belgium; 3 VIB Center for Cancer Biology, Leuven, Belgium; 4 Laboratory for Translational Genetics, Department of Human Genetics, KU Leuven, Leuven, Belgium; 5 Department of Development and Regeneration, KU Leuven, Leuven, Belgium; 6 Queen Charlotte’s and Chelsea Hospital, Imperial College, London, UK; 7 Department of Oncology, Laboratory of Tumor Immunology and Immunotherapy, Immunovar Research Group, KU Leuven, Leuven, Belgium; 8 Department of Obstetrics and Gynecology, Innsbruck Medical University, Innsbruck, Austria; 9 Department of Gynecology, Campus Virchow, Charité, Universitätsmedizin Berlin, Freie Universität Berlin, Humboldt-Universität zu Berlin, and Berlin Institute of Health, Berlin, Germany; 10 National Center for Tumor Diseases (NCT), Dresden, Germany; 11 German Cancer Consortium (DKTK), Dresden, Germany; 12 Department of Gynecology and Obstetrics, University Hospital Carl Gustav Carus Dresden, TU Dresden, Dresden, Germany; 13 Department of Metabolism, Digestion and Reproduction, Imperial College London, London, UK

**Keywords:** Ovarian cancer, Diagnostic markers

## Abstract

Fragmentation patterns of plasma cell-free DNA (cfDNA) are known to reflect nucleosome positions of cell types contributing to cfDNA. Based on cfDNA fragmentation patterns, the deviation in nucleosome footprints was quantified between diagnosed ovarian cancer patients and healthy individuals. Multinomial modeling was subsequently applied to capture these deviations in a per sample nucleosome footprint score. Validation was performed in 271 cfDNAs pre-surgically collected from women with an adnexal mass. We confirmed that nucleosome scores were elevated in invasive carcinoma patients, but not in patients with benign or borderline disease. Combining nucleosome scores with chromosomal instability scores assessed in the same cfDNA improved prediction of malignancy. Nucleosome scores were, however, more reliable to predict non-high-grade serous ovarian tumors, which are characterized by low chromosomal instability. These data highlight that compared to chromosomal instability, nucleosome footprinting provides a complementary and more generic read-out for pre-surgical diagnosis of invasive disease in women with adnexal masses.

## Introduction

Because of the spatial and temporal heterogeneity present in tumors and due to comorbidities associated with obtaining tumor biopsies, conventional methods to sequentially obtain tumor tissue from cancer patients are difficult to implement in clinical practice. Cell-free DNA (cfDNA) however, obtained from the blood of cancer patients, offers a non-invasive alternative for early detection of a primary or relapsed tumor, for monitoring tumor progression or detecting resistance to cancer therapy.

Low concentrations of cfDNA are present in plasma of healthy individuals in the form of short double-stranded DNA fragments; 70–90% of this cfDNA is derived from leukocytes, while the remaining amounts originate from several other organs, such as the liver^1,2^. In cancer patients, a highly variable percentage of cfDNA originates from the tumor. Previous and ongoing efforts to characterize this tumor-specific fraction (ctDNA, i.e. circulating tumor DNA) focus on the detection of tumor-specific genetic variation, i.e. somatic mutations and copy number alterations (CNAs). However, this approach often requires a priori knowledge of the mutation spectrum of the tumor or is limited to the detection of tumors characterized by a certain degree of chromosomal instability.

In order to more generically detect ctDNA, several efforts have focused on the analysis of epigenetic features of cfDNA^3^. Tumor-specific patterns of DNA methylation have, for instance, been used to identify which tissues or cell types are contributing to the plasma cfDNA fraction^1,4–6^. Applying ChIP-seq on cell-free DNA could recently identify chromatin marks, informative of cellular gene activity in the tissue of origin^7^. Other approaches leveraged the analysis of cell-free DNA fragmentation, using whole-genome sequencing (WGS) of cfDNA to locate nucleosome positions, their occupancy and spacing in the cfDNA. Indeed, it is hypothesized that the DNA at the sites of the nucleosomes in apoptotic cells is protected at least to some extent against degradation by nucleases and that by analysing WGS data the location of the nucleosomes can be determined. Indeed, as a result of these nucleases, the average size of cfDNA is 167 bps, which corresponds to the length of a DNA fragment wrapped around a histone core (the nucleosome, ±147 bps) and its H1 linker histone (±20 bps). Further, since the genomic distribution of nucleosomes is considered to be cell-type specific^8^, mapping of cell type-specific nucleosome positions can be used to assess which tissues are contributing to cfDNA. Initial evidence for this came from studies focusing on the size distribution of cfDNA fragments using WGS^9,10^. Building on these findings, Snyder et al.^11^ demonstrated how spacing between nucleosomes can be leveraged to identify the tissue-of-origin of cfDNA. Cristiano et al.^12^ used counts of short and long fragments in 5 Mbp windows to estimate the tissue of origin. Ulz et al.^13^ analysed the sequencing depth at transcription start sites in cfDNA to infer tumor-specific gene expression, while Straver et al.^14^ used genome-wide deviations from expected nucleosome positions to quantify the percentage of fetal DNA in plasma of pregnant women.

Here, we elaborate on the latter approach and assess whether low-coverage WGS (LC-WGS) can be used to detect invasive ovarian tumors by assessing the nucleosome footprints in the cfDNA. We previously reported how chromosomal instability measured by LC-WGS of cfDNA distinguishes high-grade serous ovarian tumors from women with benign adnexal masses, but fails to reliably detect other ovarian cancer histologies characterized by less chromosomal instability^15^. We therefore explore in a large cohort of patients presenting with adnexal masses whether nucleosome footprinting of LC-WGS data is also able to detect these histologies. Moreover, we assess whether combining chromosomal instability and nucleosome footprinting in cfDNA is more reliable in detecting invasive ovarian tumors in women with an adnexal mass than either method alone.

## Results

### Plasma cfDNA fragments display a nucleosome footprint

**Fig. 1:**
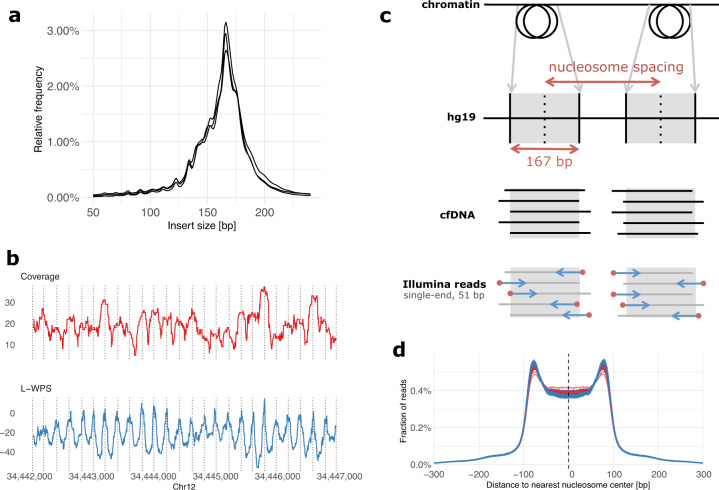
Nucleosome footprint in paired-end and single-end cfDNA sequencing data. **a** The insert size distributions of three plasma samples sequenced at high coverage using paired-end sequencing data shows fragment lengths centered on the size of nucleosome-bound DNA. **b** Coverage and L-WPS score (as defined by Snyder et al.^11^; same genomic region is displayed) based on paired-end sequencing data of one plasma sample, illustrating specific positioning of nucleosomes and their footprint in plasma cfDNA. **c** In single-end sequencing data, it is expected that mapped reads will tend to start (red dots) at the boundaries of nucleosomes. **d** When constructing a genome-wide distribution of the distances between all read start positions and the centers of the nearest expected nucleosomes as derived from a reference experiment in healthy individuals^11^, the result is an M-shaped distribution with an enrichment of read starts at the edges of nucleosomes and a depletion at the centers of nucleosomes. The distributions shown here are derived from cfDNA samples of 125 healthy individuals and 43 patients with relapsed HGSOC, shown in blue and red respectively. Compared to healthy individuals (blue), plasma samples of relapsed HGSOC patients (red) show a reduced enrichment of read starts at the nucleosome edges and a reduced depletion at nucleosome centers.

First, we confirmed that LC-WGS of cfDNA can be used to retrieve information about nucleosome positions. For this, three cfDNA samples from high-grade serous ovarian carcinoma (HGSOC) patients were selected for paired-end sequencing at high coverage. As expected, the size of cfDNA fragments corresponded to the length of DNA wrapped around histones, with a peak occurring at 167 bps (Fig. 1a). We also detected additional peaks with a length of 10 bps higher or lower, which reflects the helical pitch of the DNA molecule wrapped around the nucleosome, as previously reported^11^. To further illustrate the position of nucleosomes in specific chromosomal regions, we used the large window protection score (L-WPS score), which reflects the number of fragments spanning a 120 bps moving window minus the number of fragments with a fragment end within the 120 bps moving window^11^. When plotting L-WPS in function of chromosomal coordinates, we were indeed able to detect where nucleosomes were positioned in specific regions of the genome. Moreover, these positions closely corresponded to the nucleosome reference positions identified by Snyder et al.^11^ in the plasma of healthy individuals (indicated by vertical lines in Fig. 1b).

### HGSOC patients display a global deviation in nucleosome footprints

We next focused on deviations of cfDNA fragmentation between healthy individuals and patients diagnosed with HGSOC. Particularly, 168 cfDNA samples were obtained from 125 healthy individuals and 43 patients with relapsed HGSOC disease. The latter group of patients was selected because we detected high allelic frequencies of TP53 mutations in the cfDNA of each patient, indicating that these patients had high amounts of HGSOC-derived ctDNA in their plasma and were therefore well suited as a training set to detect HGSOC malignancy. Rather than performing WGS at full depth, we conducted LC-WGS with a median of 11.3 × 10^6^ single-end reads per sample, corresponding to a median coverage of 0.18× (see Supplementary Table 1). For every sample, reads were mapped and distances were calculated between the start of a sequencing read (i.e., a cfDNA fragment boundary) and the center of the nearest nucleosome from a reference list of 13 × 10^6^ nucleosomes (Fig. 1c). This reference list was generated from plasma of healthy individuals^11^. The distribution of these distances displayed an M-shaped curve, as shown in Fig. 1d, with proportionally more cfDNA fragments starting at the edges compared to the centers of expected nucleosome positions.

When plotting these distributions separately, either for the 125 healthy individuals or 43 cancer patients, we noticed that edges of nucleosomes were relatively enriched and centers depleted for start positions of sequencing reads from healthy individuals relative to cancer patients (Fig. 1d). This observation led us to hypothesize that nucleosome footprints in cfDNA from cancer patients deviate from the reference list of nucleosome positions constructed in plasma from healthy individuals. This reflects a shift in the distribution of cell types contributing to the circulating cfDNA pool, suggesting that it can be used as a biomarker to detect invasive disease in women with an adnexal mass.

### Calculating nucleosome and genome-wide *z*-scores based on cfDNA

Next, we explored whether based on fragmentation patterns in cfDNA, we were able to predict malignancy in a clinical cohort of patients with adnexal masses. Particularly, this cohort consisted of baseline cfDNA samples collected from 271 new patients, of which 130 exhibited on pathological examination a benign adnexal mass, 41 had a borderline ovarian tumor (BOT), 92 exhibited invasive ovarian disease, and 8 cases presented with adnexal metastases of a non-ovarian malignancy (Table 1). We performed LC-WGS on each cfDNA sample with a median of 9.8 × 10^6^ single-end reads per sample, corresponding to a median read depth of 0.16× (see Supplementary Table 1). We quantified the degree of overall deviation in cfDNA fragments using the above-described 168 LC-WGS samples as positive and negative training sets to predict malignancy. Particularly, sequencing reads were mapped and M-shaped distributions of distances between start positions of sequencing reads and nucleosome centers of a reference set were constructed (see Methods section). Nucleosome scores between 0 and 1 were calculated for each of the 271 plasma samples as described in the Methods section, where values around 0 correspond to reference healthy profiles and values around 1 correspond to reference HGSOC profiles.

**Table 1:** Clinical characteristics of the 271 patients with adnexal masses.

	Patients with an adnexal mass			
	(*n* = 271)			
	Benign mass	Borderline carcinoma	Invasive carcinoma	Metastatic tumor
	(*n* = 130)	(*n* = 41)	(*n* = 92)	(*n* = 8)
Age (in years)				
Median	53	52	64	55
IQR	43–64	37–63	57–73	52–69
Adnexal histology				
Benign				
Cystadenoma	21	–	–	–
Cystadenofibroma	52	–	–	–
Fibrothecoma	1	–	–	–
Teratoma	25	–	–	–
Leiomyoma	13	–	–	–
Other	18	–	–	–
Borderline				
Serous	–	22	–	–
Mucinous	–	15	–	–
Other	–	4	–	–
Invasive				
High-grade serous	–	–	62	–
Low-grade serous	–	–	6	–
Mucinous	–	–	8	–
Endometrioid	–	–	9	–
Clear-cell	–	–	3	–
Non-epithelial	–	–	4	–
Metastasis				
Gastric cancer	–	–	–	3
Other	–	–	–	5
FIGO stage				
IA	–	30	15	–
IB	–	3	–	–
IC	–	3	8	–
IIA	–	1	1	–
IIB	–	1	2	–
IIIA	–	2	4	–
IIIB	–	1	8	–
IIIC	–	–	22	–
IVB	–	–	32	–
CA-125 (in kU/L)				
Median	20	30	206	37
IQR	12–34	18–109	63–643	23–91

**Fig. 2:**
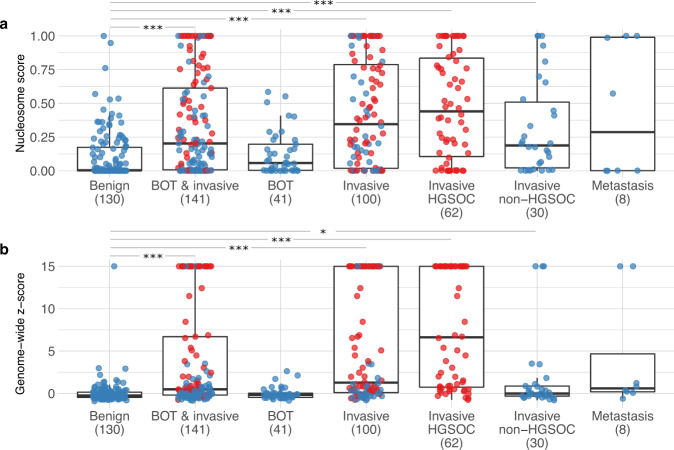
Distribution of nucleosome scores and genome-wide *z*-scores, according to histology. Nucleosome scores (**a**) and genome-wide *z*-scores (**b**) are shown for 130 patients with benign ovarian disease, 141 patients with borderline and invasive carcinoma (including 8 patients with metastases) combined, 41 patients with borderline ovarian tumors (BOT), 100 patients with invasive ovarian carcinoma (including 8 patients with metastases), 62 patients with HGSOC, 30 patients with non-HGSOC, and 8 patients with adnexal metastases of other primary cancers. Every case is indicated by a blue dot and HGSOC cases are highlighted in red. The axis of the genome-wide *z*-scores was truncated for visualization purposes. ***p-value < 0.001; *p-value < 0.05 (Mann–Whitney). Further descriptive statistics are detailed in Supplementary Tables 2 and 3.

The distribution of all nucleosome scores for patients with benign, borderline, and invasive disease is shown in Fig. 2a (see also Supplementary Table 2 and Supplementary Fig. 3 stratified for FIGO stage). Additionally, we reconstructed whole-genome copy number alteration (CNA) profiles and calculated the corresponding genome-wide *z*-scores (Fig. 2b and Supplementary Table 3)^15^. We did not observe a substantial association of sequencing depth with either nucleosome scores or genome-wide *z*-scores (see Supplementary Fig. 1). In the 130 samples from patients with benign tumors, the nucleosome score and genome-wide *z*-score decreased on average with 0.00 and 0.03 for every million sequenced reads, an effect we consider negligible. Patients with an increased body mass index (BMI) have an increased turnover of adipocytes, which may decrease the fraction of ctDNA^16^. However, we could not find a significant association between baseline BMI values and either nucleosome scores or genome-wide *z*-scores (see Supplementary Fig. 2). For every unit increase of BMI, the scores of patients with a benign tumor decreased on average with 0.00 and 0.11 respectively. Thus, we assume that both sequencing depth and BMI do not substantially affect the results of our cfDNA analyses.

### Clinical correlations of nucleosome scores and genome-wide *z*-scores

Next, we explored how these nucleosome and genome-wide *z*-scores correlated with clinical characteristics of the 271 patients. Overall, we observed low values for nucleosome and genome-wide *z*-scores in patients with benign disease (Fig. 2a, b). As with genome-wide *z*-scores, nucleosome scores of borderline carcinomas did not differ from patients with benign disease. On the contrary, advanced-stage (FIGO IV) cases displayed a very high median genome-wide *z*-score of 16.5 (*n* = 32; Supplementary Table 3). This also applied to the nucleosome scores, which with a median score of 0.65 was highest in advanced-stage FIGO IV patients. Overall, the median nucleosome score for all patients with invasive disease was 0.35, while for BOTs and benign tumors the median score was respectively 0.06 and 0.00.

We previously reported how genome-wide *z*-scores were not elevated in patients with invasive ovarian cancer that did not present with a high-grade serous histology (hereafter referred to as non-HGSOC patients), including non-epithelial histology^15^. In the current study, we again observed an increased genome-wide *z*-score in only 5 out of 30 non-HGSOC patients. Probably, lack of genomic instability in non-HGSOC patients explains why, compared to HGSOC patients, these exhibit a weaker performance for the genome-wide *z*-score. Remarkably, however, we did observe a substantial increase in nucleosome scores in these 30 non-HGSOC tumors (Fig. 2a): the median nucleosome scores were 0.07, 0.33, 0.09, 0.19, and 0.07 for patients with clear cell, endometrioid, low-grade serous, mucinous, and non-epithelial non-HGSOC disease (Supplementary Table 2). Overall, this suggests that nucleosome footprinting may be useful for the detection of tumors not characterized by CNAs. As such, nucleosome and genome-wide *z*-scores, which can both be derived from the same LC-WGS data, can possibly provide independent diagnostic information.

### Performance of nucleosome-based prediction of malignancy

**Fig. 3:**
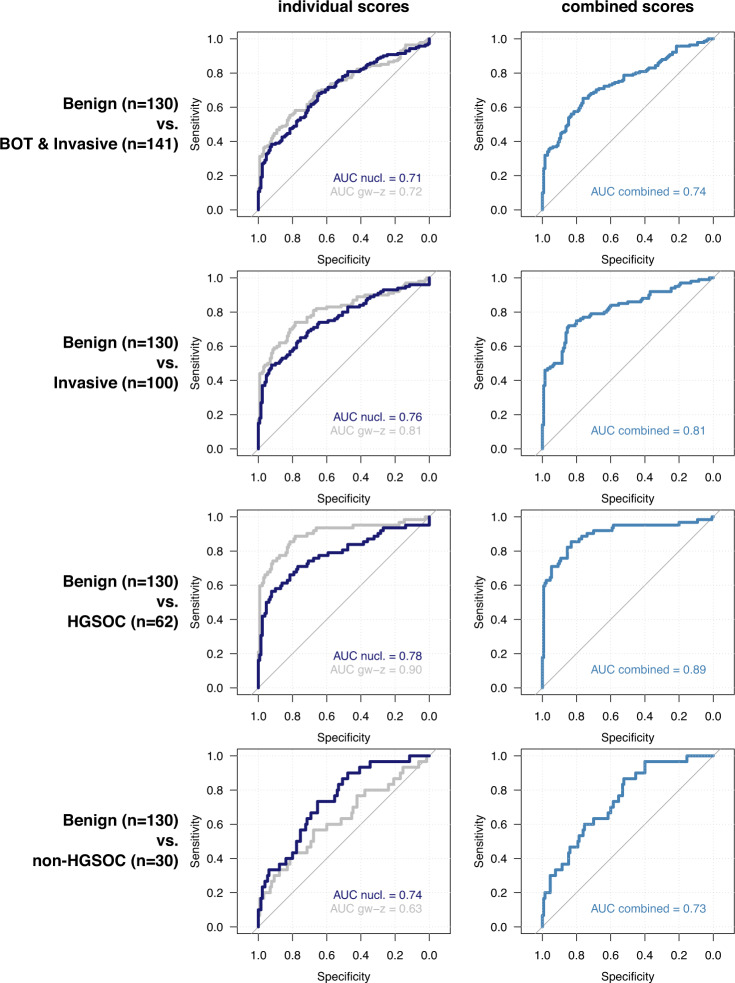
ROC analysis. ROC curves for nucleosome scores (“nucl.”) and genome-wide *z*-scores (“gw-*z*”) to discriminate patients with benign ovarian disease (*n* = 130) from patients with borderline (BOT) and invasive carcinoma (*n* = 141, including 8 patients with metastases; first row); patients with invasive carcinoma (*n* = 100; second row); patients with HGSOC disease (*n* = 62; third row); patients with non-HGSOC disease (*n* = 30; fourth row). ROC curves for nucleosome and genome-wide *z*-scores were then combined in a single predictor and the optimism-corrected AUC value was calculated (second column).

In order to further evaluate whether nucleosome or genome-wide *z*-scores can be used to predict malignancy in women with adnexal masses, we generated ROC curves and calculated AUC values to determine specificities and sensitivities of both scores (Fig. 3). Nucleosome and genome-wide *z*-scores could distinguish 130 benign cases from a combined group of 141 patients with BOT, invasive carcinoma and ovarian metastasis, displaying an AUC value of 0.71 (95% CI: 0.65–0.77) and 0.72 (95% CI: 0.66–0.78) for both scores, respectively (Fig. 3). When combining both metrics in a single ROC curve (see Methods), AUC values improved to 0.74 (95% CI: 0.68–0.80). When only invasive carcinoma was considered relative to benign cases (i.e., excluding BOTs), AUC values increased to 0.76 (95% CI: 0.70–0.82) and 0.81 (95% CI: 0.75–0.87) for nucleosome and genome-wide *z*-scores respectively (Fig. 3) and to 0.81 (95% CI: 0.76–0.87) when both scores were combined. Supplementary Figure 4 shows the metrics for discrimination of BOTs and metastatic disease and Supplementary Fig. 5 shows the metrics for invasive disease, stratified for FIGO stage. Additionally, AUC values of both metrics to discriminate HGSOC cases (*n* = 62; all FIGO stages) from benign cases (*n* = 130) were respectively, 0.78 (95% CI: 0.70–0.86) and 0.90 (95% CI: 0.84–0.95), respectively, or 0.89 (95% CI: 0.84–0.95) when both scores were combined (Fig. 3). The latter results confirm the value of assessing chromosomal instability in cfDNA for the detection of tumors with large-scale CNAs. Indeed, to detect HGSOC in cfDNA the genome-wide *z*-score exhibited superior values compared to the nucleosome score^15,17^.

We also explored the predictive value of pre-treatment serum CA125 levels, which showed a good predictive value across the different comparisons (Supplementary Fig. 6). For the detection of HGSOC versus benign disease, CA125 performed equally well as genome-wide z-score testing (AUC 0.92 versus 0.90 respectively) and better than nucleosome score (AUC 0.78). Regarding non-HGSOC disease, CA125 performed equally well as the nucleosome score (AUC 0.75 versus 0.74 respectively) and better than genome-wide *z*-score (AUC 0.63).

As we previously observed that a significant number of non-HGSOC cases, which typically are characterized by low genome-wide *z*-scores (see below), exhibit elevated nucleosome scores, we also assessed how both tests performed when comparing non-HGSOC cases (*n* = 30; all FIGO stages) to benign cases (*n* = 130). Nucleosome scores performed better than genome-wide *z*-scores (AUC 0.74 (95% CI: 0.65–0.84) versus 0.63 (95% CI: 0.51–0.75) respectively), illustrating that a subset of cases with a low genome-wide *z*-score (typically non-HGSOC cases) might be detectable through an independent nucleosome-based analysis of LC-WGS data (Fig. 3). Notably, by comparing AUC values of both HGSOC and non-HGSOC cases to patients with benign disease using the nucleosome (0.78 versus 0.74, respectively) and genome-wide z-score (0.90 versus 0.63, respectively), the sensitivity of the nucleosome score appeared stable across both HGSOC and non-HGSOC subgroups, indicating it to be a more generic test to detect tumor-derived cfDNA.

**Fig. 4:**
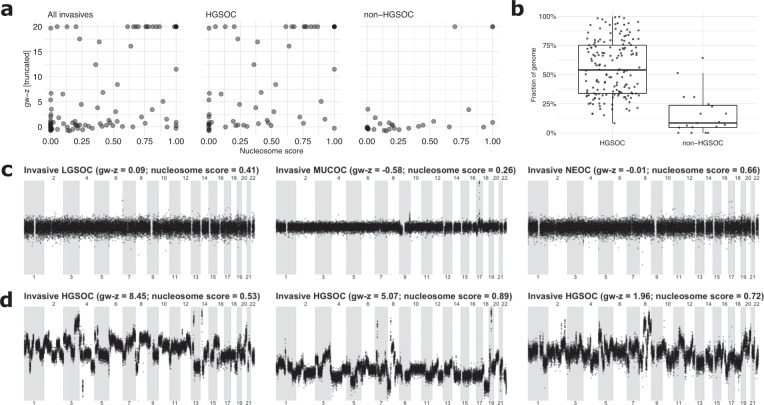
Characteristics of non-HGSOC cases. **a** Correlation between nucleosome and genome-wide *z*-scores for all invasive tumor samples (including eight metastasis samples), HGSOC and non-HGSOC samples. **b** Fraction of the genome that is not copy-neutral for a HGSOC^30^ and non-HGSOC cohort. **c**, **d** Illustrations of genomic representation profiles obtained from baseline fresh-frozen tumor tissue for three non-HGSOC samples (LGSOC, MUCOC, and NEOC) and for three HGSOC samples.

Next, we correlated genome-wide *z*-scores and nucleosome scores for all invasive cases (*n* = 100, including 8 patients with a non-ovarian primary tumor with a metastasis to the ovary) and for both subgroups of HGSOC (*n* = 62) and non-HGSOC cases (*n* = 30). Although both scores were significantly correlated in general (Spearman’s rho = 0.58; *p*-value < 0.05), this correlation was less pronounced in the non-HGSOC subgroup (Fig. 4a; Spearman’s rho = 0.64 for HGSOC and 0.47 for non-HGSOC). By visually inspecting the plots, we noticed a number of patients (*n* = 11) with an elevated nucleosome score (>0.25) but a baseline genome-wide *z*-score in the non-HGSOC subgroups (Fig. 4a and Supplementary Fig. 7). Vice versa, only one patient presented with a low nucleosome (<0.25) but high (>2.5) genome-wide *z*-score. One patient had a high nucleosome and genome-wide *z*-score, while 17 patients had both a low nucleosome and genome-wide *z*-score, respectively. When performing LC-WGS on 19 DNA samples that were available from matching non-HGSOC tumors, we could indeed observe low levels of genome-wide aneuploidy compared to HGSOC tumors (Fig. 4b). Figure 4c illustrates this observation for a low-grade serous (LGSOC), mucinous (MUCOC), and non-epithelial (NEOC) ovarian carcinoma sample. These profiles were different from HGSOC tumors, which generally show very high instability (Fig. 4d). The low chromosomal instability of non-HGSOC tumors was similarly reflected in a low genome-wide *z*-score in cfDNA; nevertheless, a higher proportion of these patients showed an increased nucleosome score (Fig. 4a and Supplementary Fig. 7). Particularly, for the three non-HGSOC examples, the nucleosome score exceeded 0.25 (Fig. 4d).

Interestingly, although we only assessed eight cfDNA samples from invasive cancer patients with a metastasis to the ovary, four of these exhibited a high nucleosome score and only two presented with a high genome-wide *z*-score. The sample size is insufficient for inference; however, given the fact that many cancer types display less chromosomal instability than HGSOC, these results may be indicative of the nucleosome score being a more generic method to detect tumor lesions based on cfDNA.

## Discussion

Here, we performed LC-WGS of plasma cfDNA and generated a nucleosome footprinting score, which for each cfDNA sample measures the overall deviation in nucleosome footprints compared to those observed in healthy individuals. As nucleosome patterns are cell-type specific, a high nucleosome score in a cfDNA sample likely reflects a change in the contribution of cell types to the cfDNA fraction in a patient. In cancer patients, where highly variable levels of tumor-derived DNA contribute to the cfDNA fraction, elevated nucleosome score could therefore predict the presence of a malignant tumor. In 271 cfDNA samples from patients presenting with an adnexal mass, we indeed observed higher nucleosome scores for patients with invasive disease relative to those presenting with benign or borderline disease.

Adnexal masses are very frequent, with some studies reporting a lifetime risk of 5–10% for women to undergo surgery for a suspected ovarian malignancy^18^. Typically, during follow-up of these adnexal masses, gynaecologists are confronted with a diagnostic dilemma, as they need to carefully balance the disadvantage of undergoing surgery (i.e., risk of complications, loss of fertility, and health-economic considerations) against the risk of missing the diagnosis of an invasive tumor. Since sequentially and invasively obtaining tumor biopsies from adnexal masses to assess potential malignancy is not a clinical option, there is a need to develop non-invasive biomarkers that could differentiate between benign versus malignant adnexal masses. Numerous efforts to develop such biomarkers have already been made. For instance, the ADNEX risk model developed by the International Ovarian Tumor Analysis (IOTA) group estimates the probability that an adnexal mass is benign, borderline, stage I cancer, stage II-IV cancer, or secondary metastatic cancer based on clinical and ultrasound data^19^. This model currently represents a clinical standard to predict ovarian malignancy, but as cfDNA-based tests are gaining momentum in clinical practice, an emerging question is whether existing predictive models could be further improved by implementing additional cfDNA-based tests.

Deep sequencing of cfDNA and subsequent size distribution analyses have provided new insights in the biology of cfDNA^11,20,21^. For instance, it was shown that cfDNA fragments originate from nucleosome-bound DNA, which is protected from degradation by nucleases. Although genomic nucleosome positions are highly dynamic, it appears that the overall nucleosome landscape is specific for each cell type, cell state and tissue^22,23^. Consequently, we can use nucleosome footprints in cfDNA to quantify the contribution of each tissue to the cfDNA. For instance, using 76 expression sets of human cell lines and tissues as a reference, Snyder et al. were able to demonstrate that tumor tissue contributes to cfDNA in 5 selected patients with advanced-stage cancer^11^. A similar approach was used in the context of prenatal diagnosis, where a different cfDNA fragmentation pattern between maternal and fetal-derived cfDNA was leveraged to calculate the fraction of fetal DNA in cfDNA from pregnant women^14^. In this study, we centered single-end sequencing reads derived from LC-WGS on a map of reference nucleosome positions and we observed that the distribution of the start positions of each read differed between a reference set of healthy individuals and a cohort of relapsed HGSOC patients with high ctDNA load. This suggests that a deviation in nucleosome footprints, associated with the presence of an invasive carcinoma, can be inferred from cfDNA-based LC-WGS data. When assigning nucleosome scores, which reflect a numeric read-out of this deviation, to each sample from a large cohort of 271 cfDNA samples obtained from patients with adnexal masses, we indeed found that the nucleosome score was elevated in patients with a malignancy compared to those with a benign lesion. Interestingly, we previously reported how chromosomal instability distinguishes HGSOC from women with benign adnexal masses using LC-WGS^15^. Compared to the genome-wide *z*-score, which was similarly increased in patients with a malignancy, the nucleosome score had a weaker performance. However, we previously also demonstrated that the genome-wide *z*-score fails to reliably detect other ovarian cancer histologies characterized by less chromosomal instability. Indeed, in non-HGSOC patients, the performance of the genome-wide *z*-score dropped considerably. The nucleosome score, however, performed better to identify non-HGSOC patients. This is a quite interesting observation as both the nucleosome and genome-wide *z*-score can be derived from the same LC-WGS data. As such, LC-WGS of cfDNA represents a single diagnostic test that has the potential to generate two independent and complementary diagnostic read-outs.

As mentioned, the nucleosome score quantifies a shift from the average cfDNA patterns of healthy individuals. These shifts most likely reflect the contribution of other tissues to the cfDNA pool in plasma. However, it is agnostic to which cell types are causing the deviation. As such, we are unable to investigate whether the deviation in nucleosome footprints is caused by tumor-derived cfDNA or whether the deviation is possibly also caused by other non-tumoral cells contributing cfDNA to the plasma. Indeed, in cancer patients there is also a major shift in the abundance and type of circulating immune cells. Changes in the levels of various circulating leukocytes have for instance been observed in ovarian cancer patients, while moreover, these changes are of important prognostic relevance^24^. Additionally, patients with other disease, such as autoimmune disease patients (e.g., lupus or multiple sclerosis) or patients with liver disease, a myocardial infarction or a kidney transplantation may also be characterized by a different composition of cell types contributing to the cfDNA^1^, which may be reflected in the nucleosome footprint because of differences in chromatin landscapes between these cell types^11^.

Based on our observations, several questions emerge. An interesting question is how to integrate the genome-wide z-score and nucleosome score in a potential clinical setting. Ultrasonography and serum CA125 testing are capable of correctly distinguishing most HGSOC tumors from benign cysts, but often additional confirmation is needed. Hence, there could be a diagnostic opportunity for both scores in combination with ultrasonography and serum CA125 testing. As such, prediction models such as the ADNEX risk model, which combines ultrasound and clinical variables, could be extended with cfDNA-based scores. Additional research, however, is needed to determine how these scores should be integrated in the current ADNEX model and how this will add to the predictive power of the ADNEX model. In addition, it remains to be investigated how different sets of control samples will affect the scores and their performances. Indeed, when using different control sets, scores may deviate, possibly leading to different risk estimates. Such heterogeneity is undesirable, and efforts may be required to control for this. Another question is related to increasing the signal-to-noise ratio of the nucleosome score that we developed. Indeed, we pooled genomic regions and assessed the average deviation of nucleosome patterns across the entire genome. We anticipate, however, that focusing the score on genomic regions specifically altered in HGSOC or non-HGSOC could still improve the performance. Our training dataset only consisted of HGSOC samples. Due to the relative low incidence of non-HGSOC cases, cfDNA data was only obtained from 30 cases of non-HGSOC tumors and these were only used in the validation dataset. This may have impacted the performance of the nucleosome score. Additional datasets and more in-depth bio-informatics analyses are needed to explore this in future work. Technical improvements such as higher sequencing coverage or paired-end sequencing, could also still contribute to an overall improved performance.

In conclusion, we here show that LC-WGS generates two biomarker read-outs that yield complementary diagnostic information. Particularly, we confirm that the genome-wide z-score efficiently detects chromosomal instability of HGSOC tumors in plasma cfDNA, while additionally, we show that non-HGSOC patients are often missed when using the genome-wide *z*-score. The latter patients may, however, be more effectively detected using nucleosome footprinting of cfDNA.

## Methods

### Ethics approval and consent to participate

Approved by the Ethics Committee Research UZ/KU Leuven (study numbers: S51375, S59207, S64035, and S64205). All included patients provided written informed consent.

### Discovery set

We collected 125 blood samples from healthy female individuals as negative controls, as approved by the Ethics Committee Research UZ/KU Leuven (study numbers: S64035 and S64205). All individuals provided written informed consent. This group consisted of healthy donors and of patients consulting the hospital for non-ovarian related gynecological complaints; the latter were only included after transvaginal ultrasound demonstrating two normal ovaries. Their median age was 52 years.

Additionally, we included plasma samples from 43 patients with relapsed HGSOC. These patients all participated in the phase 2 GANNET53 trial^25^ (NCT02012192). This trial included female patients with platinum-resistant relapsed ovarian cancer, treated with paclitaxel with or without the Hsp90-inhibitor ganetespib. Prospective collection of baseline blood samples for cfDNA extraction before treatment was included in the study protocol. In this manuscript, the first batch of available baseline blood samples (*n* = 43, median age 62 years) were used for cfDNA extraction and development of the model.

### Validation set

Pre-treatment blood samples were obtained from 271 patients with an adnexal mass, undergoing surgical treatment. Patients were consecutively enrolled in the TRANS-IOTA study after diagnosis with transvaginal ultrasound at the University Hospitals Leuven (Belgium) between June 2015 and February 2017 (approved by the Ethics Committee Research UZ/KU Leuven: S51375/NCT01698632 and S59207/NCT02847832). All patients provided written informed consent. Age, BMI, final histology, FIGO stage, and pre-treatment serum CA125 levels were collected from the electronic patient files. Exclusion criteria were presence of or active therapy for non-ovarian cancer at the moment of inclusion, presence of immune disease, treatment with immunomodulators, pregnancy, age below 18 years, surgery of the suspected mass elsewhere prior to inclusion and positive infectious serology (HIV, HepB, and HepC).

### Sample processing

Plasma was prepared and cfDNA was extracted as previously described^15^. DNA sequencing libraries were prepared using the KAPA DNA Library Preparation Kit (KAPA Biosystems, Wilmington, MA, USA). All samples were subjected to low-coverage whole-genome sequencing on a HiSeq platform (Illumina, San Diego, CA, USA) using a V4 flow cell generating 1 × 51 bp reads, with a median read count of 10.4 × 10^6^ reads per sample (Supplementary Table 1). For 19 of the non-HGSOC plasma samples, a matching formalin-fixed paraffin-embedded (FFPE) tumor biopsy sample was available. These were sequenced using LC-WGS similarly as to the plasma samples. In addition, three plasma samples with high tumoral load were selected for genome-wide paired-end sequencing on a NovaSeq 6000 platform (Illumina, San Diego, CA, USA), generating 2 × 151 bp reads at coverage 7.4×, 18.8×, and 30.6×.

### Bio-informatics pipeline

Raw sequencing reads were mapped to the human reference genome Hg19 using BWA v0.7.1^26^. Duplicate and low-quality reads were removed by Picard Tools v1.11 and Samtools v0.1.18 respectively^27^.

#### Genome-wide *z*-score

Chromosomal instability was assessed using genome-wide *z*-score calculation, as described previously^15^. Briefly, the genome was divided in 1000 kbp bins, excluding sex chromosomes. Reads were counted in each bin and adjusted for total number of reads, GC-content and mappability. The bin values were smoothened by taking moving window averages of 50 adjacent bins, and then *z*-scores were calculated for each window using the distribution of healthy individuals as a reference. Subsequently, a single genome-wide *z*-score was calculated for each sample as the *z*-score (again using healthy individuals as a reference) of the sum of squares of all window *z*-values.

#### Nucleosome score

Genome-wide deviation of nucleosome footprints was quantified in cfDNA using a nucleosome score. To this end, we compared the start positions of 51 bp Illumina reads—representing the boundaries of circulating cfDNA fragments—to a map of nucleosome positions found in plasma of healthy individuals. We used a previously published list of 13 × 10^6^ nucleosome positions as a reference^11^. We calculated distances on autosomes between each read start and the nearest nucleosome center from the reference list. We only focused at distances *i* within a [−300, +300] bp range, and counted their frequencies *y*_*i*_. The distribution of distances displays a typical M-shaped profile: read starts are enriched at the edges of nucleosomes and are depleted at the centers of nucleosomes^14^.

To quantify deviations of this profile, we trained a model using plasma samples of a training set of 125 healthy individuals and 43 relapsed HGSOC patients. Given these reference samples *j*, the frequencies of distances *i* within the [−300, +300] range are modeled as a multinomial stochastic variable:

$$y_j\sim {{{\mathrm{Multinomial}}}}(\theta = \theta _k),$$

in which *y*_*j*_ is a vector for sample *j* containing the observed number of read starts at distances *i* from −300 to +300, and *θ*_*k*_ represents a probability simplex containing the probabilities for all distances *i* given class *k*_*j*_ of the sample (either healthy or HGSOC, depending on training sample *j*). As such, *θ*_HGSOC_ and *θ*_healthy_ represent how read starts are positioned around expected nucleosome centers for samples of both classes.

After this training step, we quantified the nucleosome score of an unknown sample using a mixture parameter λ which optimizes the probability simplex *θ*_mixt_ as a weighted average of *θ*_HGSOC_ and *θ*_healthy_ given the observed read counts *y*_obs_:

$$\theta _{{\mathrm{mixt}}} = \lambda \theta _{{\mathrm{HGSOC}}} + (1{{{\mathrm{ }}}} - \lambda )\theta_{{\mathrm{healthy}}},$$

$$y_{{\mathrm{obs}}}\sim {{{\mathrm{Multinomial}}}}(\theta = \theta _{{\mathrm{mixt}}}).$$

If the M-shaped profile of a sample corresponds closely to those of the samples in the HGSOC reference set, *λ* will have an estimated value near 1; if the M-shaped profile corresponds closely to the healthy reference samples, the value of *λ* will be estimated to be near 0.

We implemented this analysis as a Bayesian hierarchical model with uninformative uniform priors in Stan (using the interface from R with package rstan v2.18.1^28^). Four parallel Markov chains of 300 iterations are run after a warm-up of 300 iterations to estimate λ. Convergence was obtained for each sample according to the Rhat statistic and a visual check of the 4 Markov chains. The nucleosome score is determined as the median of the posterior sample of *λ*, which is constrained within 0 and 1.

#### Non-HGSOC tumor tissue

In all, 19 FFPE tumor tissues, matched to a non-HGSOC plasma sample, were mapped to the human reference genome and reads were counted in bins in the same way as described above for the plasma samples. ASCAT^29^ was then used to estimate copy number segments for these tumors. The lengths of segments with non-neutral copy number were summed and expressed as a fraction of the total segment lengths. As a comparison, this was plotted against the same fractions in a published cohort of HGSOC tumor samples^30^.

### Statistical analysis

Boxplots were plotted where the lower and upper hinges represent the first and third quartile; the whiskers extend to maximum 1.5 times the interquartile range from the hinge. All individual points are plotted on top of boxplots, with random noise added in horizontal direction to visualize overlapping points. Mann-Whitney test was used to compare cohorts. Receiver operation characteristic (ROC) curves were constructed and the corresponding area under the curve (AUC) values were calculated using the pROC package v1.17.0.1 in R^31^. To combine genome-wide *z*-scores and nucleosome scores into a single predictor and corresponding ROC curve, a logistic regression model with ranks of both scores was fitted. The optimism of the AUC value of the combined predictor was estimated using 500 non-parametric bootstrap iterations and subtracted to obtain an unbiased estimate of performance^32^. All data was processed in R version 3.1.3^33^. GNU parallel was used for running scripts in parallel^34^.

### Reporting summary

Further information on research design is available in the Nature Research Reporting Summary linked to this article.

## Supplementary information

Supplementary material

Reporting summary

## Data Availability

Low-coverage whole-genome sequencing data of the 271 patients and 125 healthy individuals have been deposited at the European Genome-phenome Archive (EGA) under study no. EGAS00001005361. Requests for accessing raw sequencing reads will be reviewed by the UZLeuven-VIB data access committee. Any data shared will be released via a Data Transfer Agreement that will include the necessary conditions to guarantee protection of personal data (according to the European GDPR law). Datasets from Snyder et al.^11^ and from Despierre et al.^30^ were used as reference dataset and comparative dataset, respectively.

## Code availability

The bio-informatics pipelines and software packages (with version number) used for data analysis have been described in the Methods.
