# Supplementary material for: Nucleosome footprinting in plasma cell-free DNA for the pre-surgical diagnosis of ovarian cancer
Source: NPJ Genom Med. 2022 Apr 28;7:30. doi: 10.1038/s41525-022-00300-5 (PMC9050708; doi:10.1038/s41525-022-00300-5)
Supplement: Supplementary file 1 — Supplementary material [file 41525_2022_300_MOESM1_ESM.pdf]

SUPPLEMENTARY FIGURES & TABLES

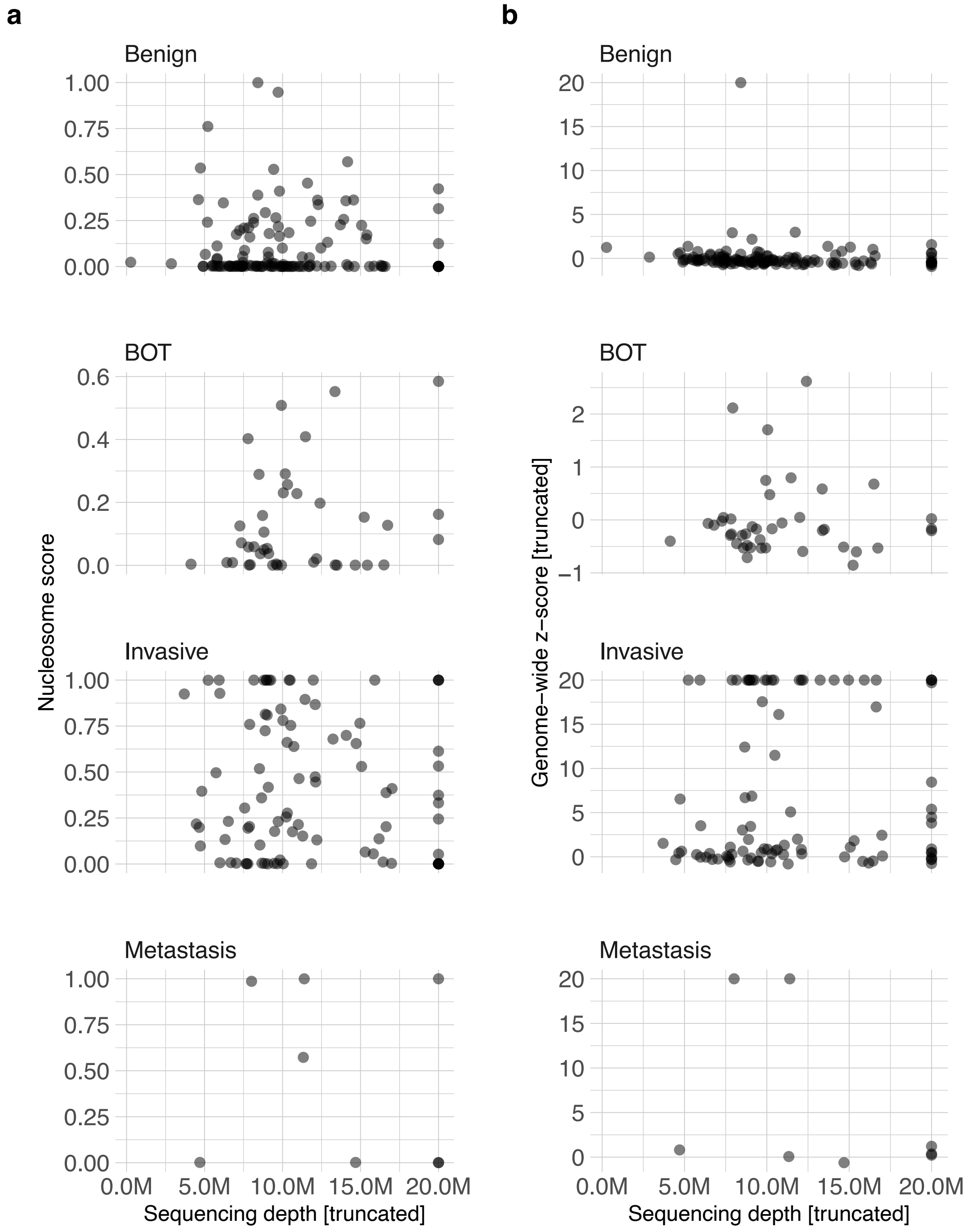

Supplementary Figure 1.

Comparison of (**a**) nucleosome scores and (**b**) genome-wide *z*-scores to the samples' sequencing depth. The axes of the genome-wide *z*-scores and sequencing depth were truncated for visualization purposes.

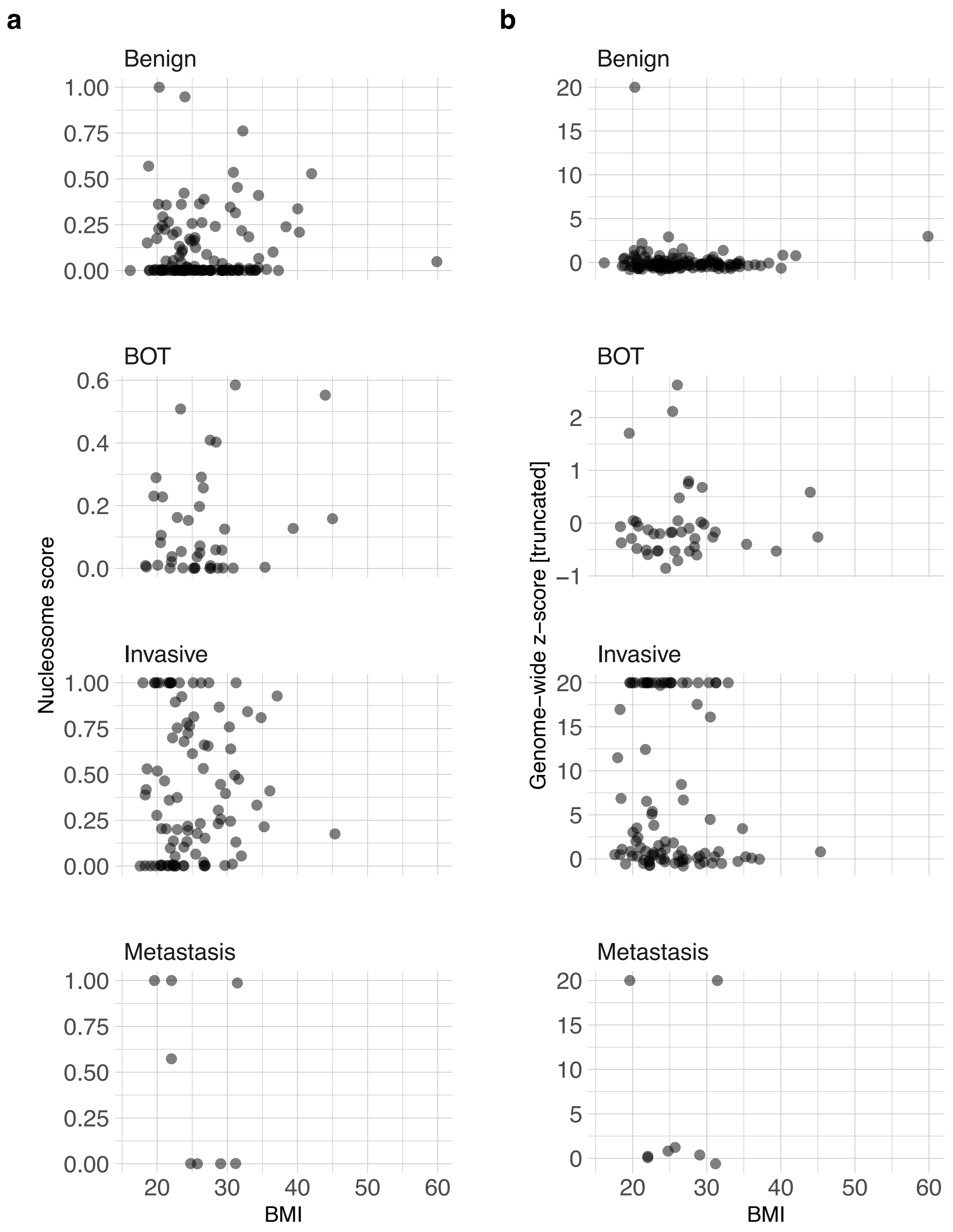

Supplementary Figure 2.

Comparison of (**a**) nucleosome scores and (**b**) genome-wide *z*-scores to the patients' BMI at the time of cfDNA sampling. The axis of the genome-wide *z*-scores was truncated for visualization purposes.

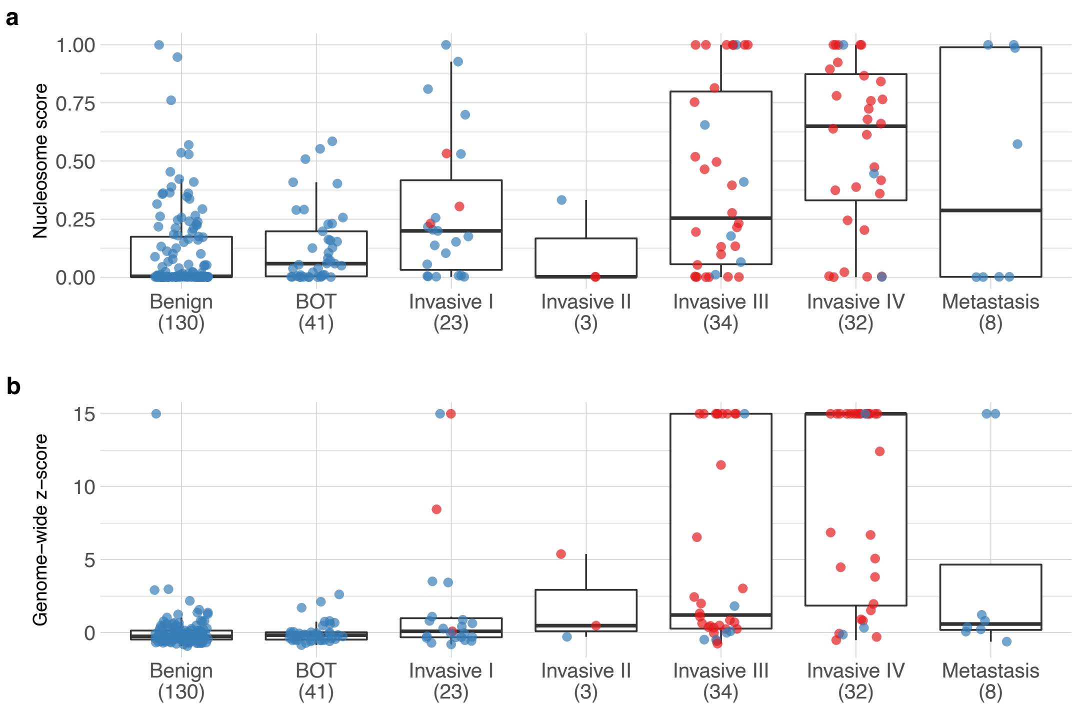

Supplementary Figure 3.

Distribution of nucleosome scores (a) and genome-wide *z*-scores (b), according to FIGO stage. Scores are shown for 130 patients with benign ovarian disease, 41 patients with borderline ovarian tumors (BOT), 92 patients with invasive ovarian carcinoma according to FIGO stage (Invasive, stage I, II, III and IV) and 8 patients with adnexal metastases of other primary cancers. Every case is indicated by a blue dot and HGSOC cases are highlighted in red. The axis of the genome-wide *z*-scores was truncated for visualization purposes. Further descriptive statistics are detailed in Supplementary Table 2 and 3.

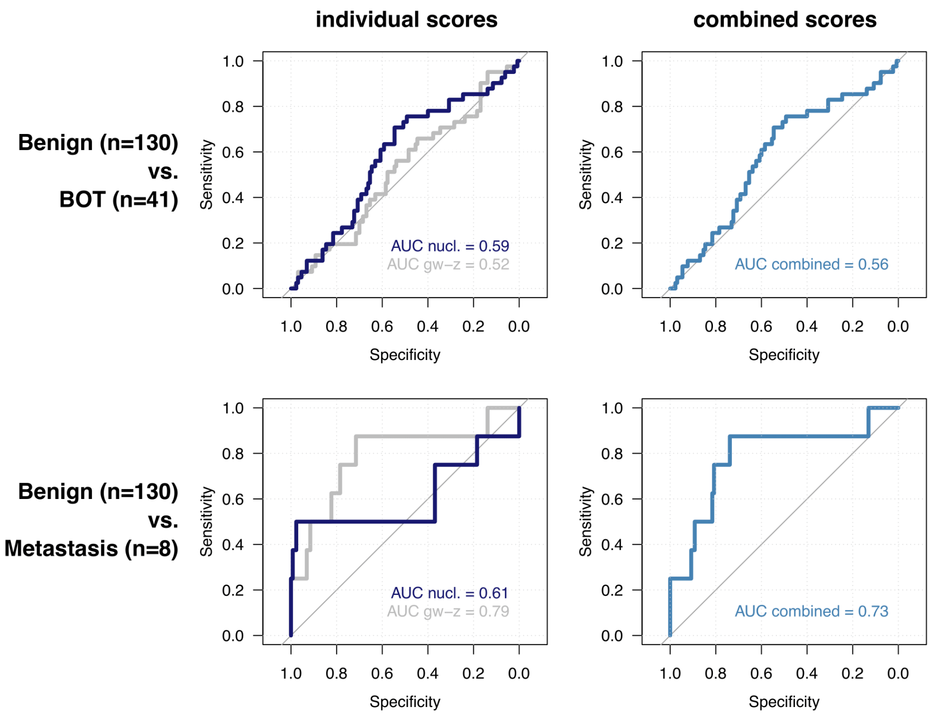

Supplementary Figure 4.

ROC curves for nucleosome scores ("nucl.") and genome-wide *z*-scores ("gw-*z*") to discriminate patients with benign ovarian disease (n=130) from patients with borderline (BOT) carcinoma (n=41); first row); patients with metastasis (n=8; second row). ROC curves for nucleosome and genome-wide *z*-scores were then combined in a single predictor and the optimism-corrected AUC value was calculated (second column).

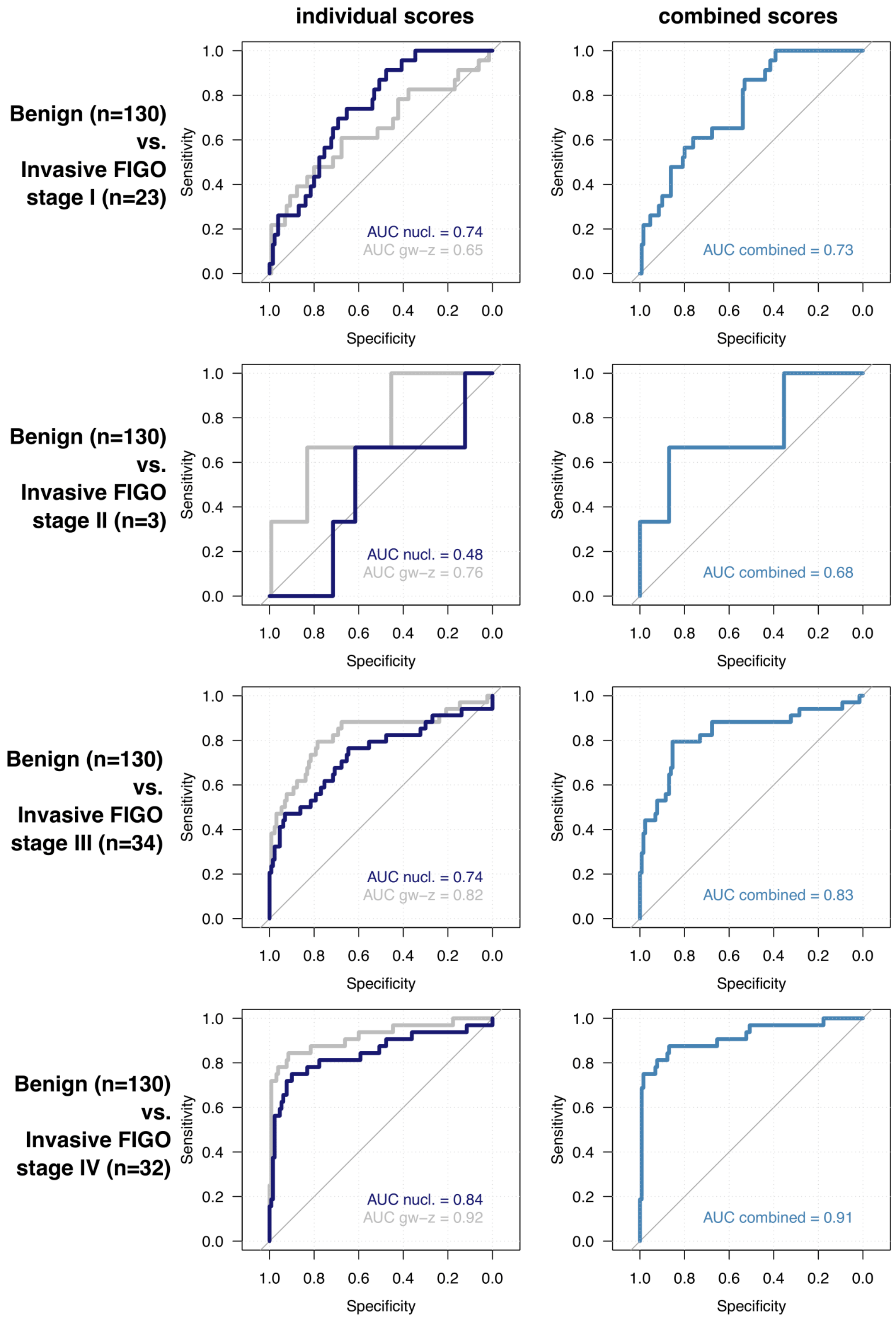

Supplementary Figure 5.

ROC curves for nucleosome scores ("nucl.") and genome-wide *z*-scores ("gw-z") to discriminate patients with benign ovarian disease (n=130) from patients with invasive carcinoma FIGO stage I (n=23; first row); patients with invasive carcinoma FIGO stage II (n=3; second row); patients with invasive carcinoma FIGO stage III (n=34; third row); patients with invasive carcinoma FIGO stage IV (n=32; fourth row). ROC curves for nucleosome and genome-wide *z*-scores were then combined in a single predictor and the optimism-corrected AUC value was calculated (second column).

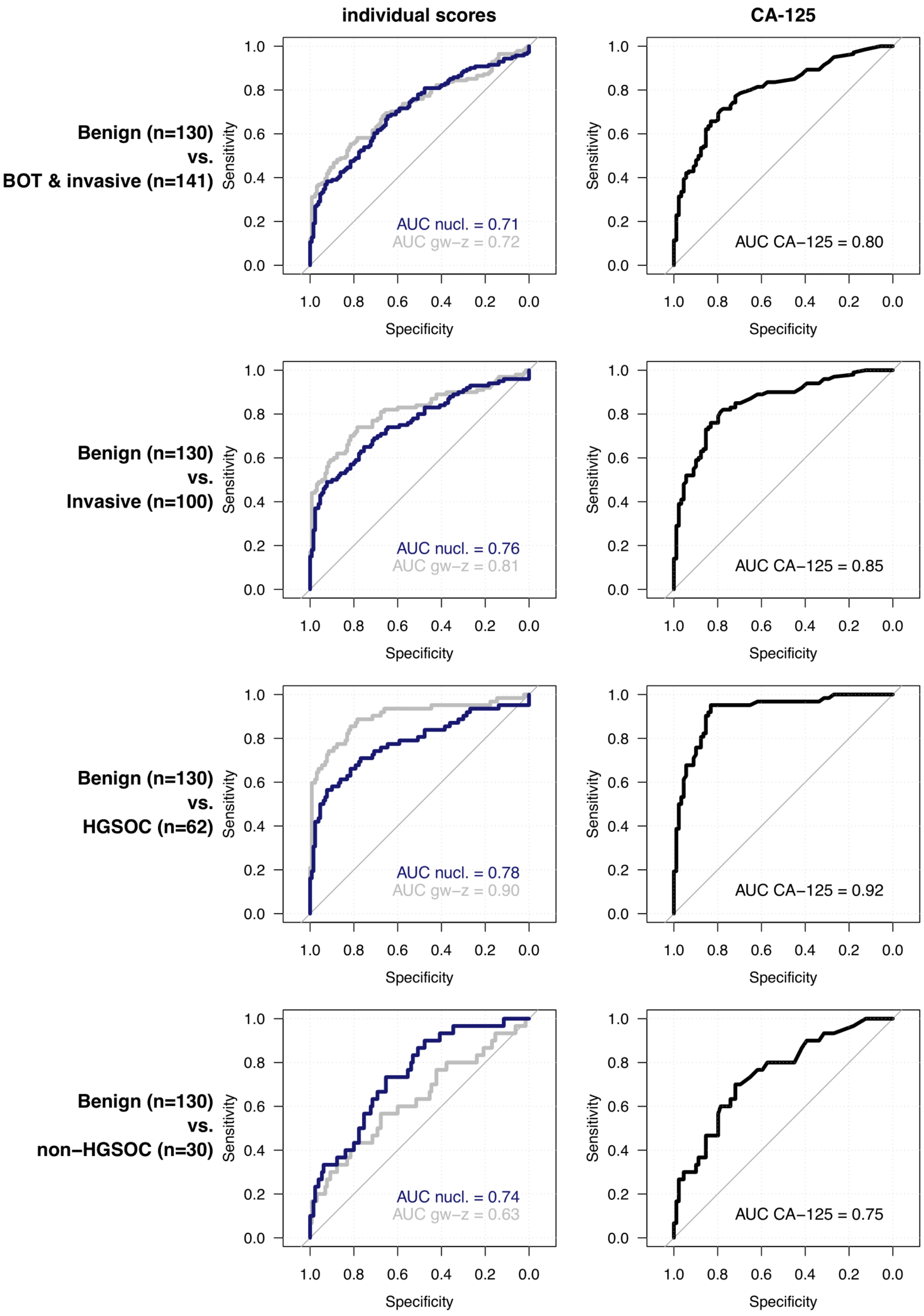

Supplementary Figure 6.

ROC curves for nucleosome scores ("nucl.") and genome-wide *z*-scores ("gw-*z*") (left column) and for CA125 levels (right column) to discriminate patients with benign ovarian disease (n=130) from patients with borderline (BOT) and invasive carcinoma (n=141, including 8 patients with metastases; first row); patients with invasive carcinoma (n=100; second row); patients with HGSOC disease (n=62; third row); patients with non-HGSOC disease (n=30; fourth row).

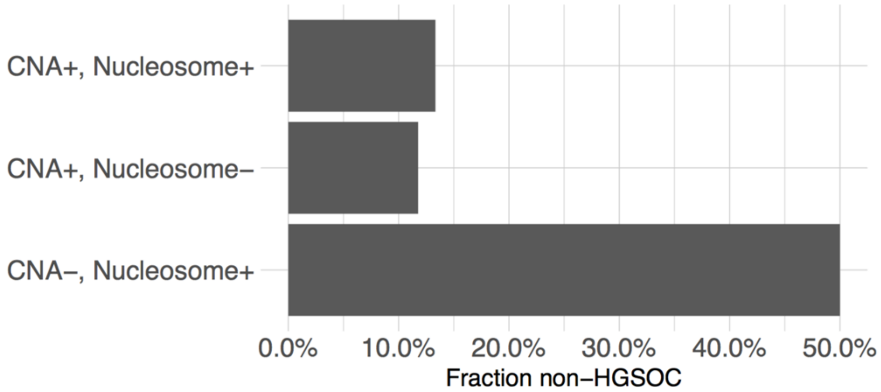

Supplementary Figure 7.
We divided the 92 samples of patients with invasive carcinoma as positive or negative for each score by taking as a threshold the 95th percentile of these scores in the subpopulation with benign tumors. The subset of samples negative for genome-wide *z*-score but positive for nucleosome score (“CNA-, Nucleosome+”) is highly enriched in non-HGSOC histologies compared to the subsets positive for genome-wide *z*-scores.

Supplementary Table 1. Number of raw sequencing reads and sequencing coverage.

	**Number of sequencing reads** Median (IQR) [million]	**Coverage** Median
**Reference samples**		
Healthy individuals (n=125)	10.8 (5.91–14.8)	0.17×
Relapsed HGSOC patients (n=43)	12.8 (11.2–15.4)	0.20×
**Patients with adnexal mass (n=271)**	9.8 (7.8–13.8)	0.16×
Benign mass (n=130)	9.5 (7.4–12.4)	0.15×
Borderline carcinoma (n=41)	9.9 (8.5–13.4)	0.16×
Invasive carcinoma (n=92)	10.3 (8.5–15.4)	0.16×
Metastasis (n=8)	13.0 (10.5–27.8)	0.21×

Supplementary Table 2. Nucleosome scores according to stage and histology.

	All patients	Stage I	Stage II	Stage III	Stage IV
**Benign mass**	(n=130)	-	-	-	-
	**median: 0.00**	**-**	**-**	**-**	**-**
	IQR: 0.00-0.17	-	-	-	-
	range: 0.00-1.00	-	-	-	-
**Carcinoma**	(n=141)	(n=59)	(n=5)	(n=37)	(n=32)
	**median: 0.20**	**median: 0.10**	**median: 0.00**	**median: 0.23**	**median: 0.65**
	IQR: 0.01-0.61	IQR: 0.01-0.23	IQR: 0.00-0.33	IQR: 0.05-0.75	IQR: 0.33-0.87
	range: 0.00-1.00	range: 0.00-1.00	range: 0.00-0.51	range: 0.00-1.00	range: 0.00-1.00
**Borderline carcinoma**	(n=41)	(n=36)	(n=2)	(n=3)	-
	**median: 0.06**	**median: 0.06**	**median: 0.25**	**median: 0.23**	**-**
	IQR: 0.00-0.20	IQR: 0.00-0.16	IQR: 0.13-0.38	IQR: 0.11-0.41	-
	range: 0.00-0.58	range: 0.00-0.55	range: 0.00-0.51	range: 0.00-0.58	-
**Invasive carcinoma**	(n=92)	(n=23)	(n=3)	(n=34)	(n=32)
	**median: 0.35**	**median: 0.20**	**median: 0.00**	**median: 0.25**	**median: 0.65**
	IQR: 0.05-0.77	IQR: 0.03-0.42	IQR: 0.00-0.17	IQR: 0.06-0.80	IQR: 0.33-0.87
	range: 0.00-1.00	range: 0.00-1.00	range: 0.00-0.33	range: 0.00-1.00	range: 0.00-1.00
**Invasive carcinoma HGSOC**	(n=62)	(n=3)	(n=2)	(n=28)	(n=29)
	**median: 0.44**	**median: 0.30**	**median: 0.00**	**median: 0.25**	**median: 0.66**
	IQR: 0.11-0.84	IQR: 0.27-0.42	IQR: 0.00-0.00	IQR: 0.04-0.86	IQR: 0.36-0.87
	range: 0.00-1.00	range: 0.23-0.53	range: 0.00-0.00	range: 0.00-1.00	range: 0.00-1.00
**Invasive carcinoma non-HGSOC**	(n=30)	(n=20)	(n=1)	(n=6)	(n=3)
	**median: 0.19**	**median: 0.16**	**median: 0.33**	**median: 0.29**	**median: 0.45**
	IQR: 0.02-0.51	IQR: 0.01-0.32	IQR: 0.33-0.33	IQR: 0.09-0.59	IQR: 0.22-0.72
	range: 0.00-1.00	range: 0.00-1.00	range: 0.33-0.33	range: 0.01-1.00	range: 0.00-1.00
**non-HGSOC: Clear cell**	(n=3)	(n=1)	-	(n=2)	-
	**median: 0.07**	**median: 0.00**	**-**	**median: 0.53**	**-**
	IQR: 0.03-0.53	IQR: 0.00-0.00	-	IQR: 0.30-0.77	-
	range: 0.00-1.00	Range: 0.00-0.00	-	range: 0.07-1.00	-
**non-HGSOC: Endometrioid**	(n=9)	(n=8)	(n=1)	-	-
	**median: 0.33**	**median: 0.46**	**median: 0.33**	**-**	**-**
	IQR: 0.20-0.81	IQR: 0.18-0.84	IQR: 0.33-0.33	-	-
	range: 0.00-1.00	range: 0.00-1.00	range: 0.33-0.33	-	-
**non-HGSOC: LGSOC**	(n=6)	(n=1)	-	(n=3)	(n=2)
	**median: 0.09**	**median: 0.01**	**-**	**median: 0.18**	**median: 0.22**
	IQR: 0.01-0.35	IQR: 0.01-0.01	-	IQR: 0.09-0.29	IQR: 0.11-0.33
	range: 0.00-0.45	range: 0.01-0.01	-	range: 0.01-0.41	range: 0.00-0.45
**non-HGSOC: Mucinous**	(n=8)	(n=7)	-	-	(n=1)
	**median: 0.19**	**median: 0.18**	**-**	**-**	**median: 1.00**
	IQR: 0.13-0.32	IQR: 0.10-0.23	-	-	IQR: 1.00-1.00
	range: 0.00-1.00	range: 0.00-0.53	-	-	range: 1.00-1.00
**non-HGSOC: Non-epithelial**	(n=4)	(n=3)	-	(n=1)	-
	**median: 0.07**	**median: 0.01**	**-**	**median: 0.66**	**-**
	IQR: 0.01-0.27	IQR: 0.01-0.07	-	IQR: 0.66-0.66	-
	range: 0.00-0.66	range: 0.00-0.14	-	range: 0.66-0.66	-
**Metastatic**	(n=8)	-	-	-	-
	**median: 0.29**	**-**	**-**	**-**	**-**
	IQR: 0.00-0.99	-	-	-	-
	range: 0.00-1.00	-	-	-	-

Supplementary Table 3. Genome-wide *z*-scores according to stage and histology.

	All stages	Stage I	Stage II	Stage III	Stage IV
**Benign mass**	(n=130)	-	-	-	-
	**median: -0.3**	-	-	-	-
	IQR: -0.5-0.1	-	-	-	-
	range: -0.9-93.1	-	-	-	-
**Carcinoma**	(n=141)	(n=59)	(n=5)	(n=37)	(n=32)
	**median: 0.5**	**median: -0.1**	**median: -0.3**	**median: 0.9**	**median: 16.5**
	IQR: -0.2-6.7	IQR: -0.4-0.6	IQR: -0.5-0.5	IQR: 0.1-21.4	IQR: 1.9-68.4
	range: -0.9-3513.6	range: -0.9-69.5	range: -0.5-5.4	range: -0.8-3513.6	range: -0.5-681.7
**Borderline carcinoma**	(n=41)	(n=36)	(n=2)	(n=3)	-
	**median: -0.2**	**median: -0.2**	**median: -0.5**	**median: -0.2**	-
	IQR: -0.5-0.0	IQR: -0.4-0.0	IQR: -0.5--0.5	IQR: -0.4--0.1	-
	range: -0.9-2.6	range: -0.9-2.6	range: -0.5--0.5	range: -0.6--0.1	-
**Invasive carcinoma**	(n=92)	(n=23)	(n=3)	(n=34)	(n=32)
	**median: 1.7**	**median: 0.1**	**median: 0.5**	**median: 1.2**	**median: 16.5**
	IQR: 0.1-23.1	IQR: -0.3-1.0	IQR: 0.1-2.9	IQR: 0.3-22.1	IQR: 1.9-68.4
	range: -0.8-3513.6	range: -0.8-69.5	range: -0.3-5.4	range: -0.8-3513.6	range: -0.5-681.7
**Invasive carcinoma HGSOC**	(n=62)	(n=3)	(n=2)	(n=28)	(n=29)
	**median: 6.6**	**median: 8.4**	**median: 2.9**	**median: 1.7**	**median: 17.0**
	IQR: 0.7-38.3	IQR: 4.3-13.0	IQR: 1.7-4.2	IQR: 0.4-26.1	IQR: 3.8-60.1
	range: -0.8-3513.6	range: 0.1-17.5	range: 0.5-5.4	range: -0.8-3513.6	range: -0.5-681.7
**Invasive carcinoma non-HGSOC**	(n=30)	(n=20)	(n=1)	(n=6)	(n=3)
	**median: -0.0**	**median: -0.0**	**median: -0.3**	**median: 0.0**	**median: 0.3**
	IQR: -0.3-0.9	IQR: -0.3-0.8	IQR: -0.3--0.3	IQR: -0.4-1.4	IQR: 0.1-76.2
	range: -0.8-811.0	range: -0.8-69.5	range: -0.3 --0.3	range: -0.5-811.0	range: -0.1-152.1
**non-HGSOC: Clear cell**	(n=3)	(n=1)	-	(n=2)	-
	**median: 1.8**	**median: 0.0**	-	**median: 406.4**	-
	IQR: 0.9-406.4	IQR: -0.0—0.0	-	IQR: 204.1-608.7	-
	range: -0.0-811.0	range: -0.0—0.0	-	range: 1.8-811.0	-
**non-HGSOC: Endometrioid**	(n=9)	(n=8)	(n=1)	-	-
	**median: 0.4**	**median: 0.4**	**median: -0.3**	-	-
	IQR: -0.3-0.9	IQR: -0.3-0.9	IQR: -0.3--0.3	-	-
	range: -0.4-69.5	range: -0.4-69.5	range: -0.3--0.3	-	-
**non-HGSOC: LGSOC**	(n=6)	(n=1)	-	(n=3)	(n=2)
	**median: -0.2**	**median: -0.3**	-	**median: -0.5**	**median: 0.1**
	IQR: -0.4-0.0	IQR: -0.3--0.3	-	IQR: -0.5--0.2	IQR: -0.0-0.2
	range: -0.5-0.3	range: -0.3--0.3	-	range: -0.5-0.1	range: -0.1-0.3
**non-HGSOC: Mucinous**	(n=8)	(n=7)	-	-	(n=1)
	**median: -0.0**	**median: -0.3**	-	-	**median: 152.1**
	IQR: -0.5-0.9	IQR: -0.6-0.5	-	-	IQR: 152.1-152.1
	range: -0.8-152.1	range: -0.8-1.1	-	-	range: 152.1-152.1
**non-HGSOC: Non-epithelial**	(n=4)	(n=3)	-	(n=1)	-
	**median: -0.1**	**median: -0.3**	**-**	**median: 0.0**	-
	IQR: -0.4-0.9	IQR: -0.5-1.6	-	IQR: -0.0--0.0	-
	range: -0.7-3.5	range: -0.7-3.5	-	range: -0.0--0.0	-
**Metastatic**	(n=8)	-	-	-	-
	**median: 0.6**	-	-	-	-
	IQR: 0.2-44.5	-	-	-	-
	range: -0.6-292.9	-	-	-	-
