# Supplementary material for: Nucleosome footprinting in plasma cell-free DNA for the pre-surgical diagnosis of ovarian cancer
Source: NPJ Genom Med. 2022 Apr 28;7:30. doi: 10.1038/s41525-022-00300-5 (PMC9050708; doi:10.1038/s41525-022-00300-5)
Supplement: Supplementary file 2 — Reporting summary [file 41525_2022_300_MOESM2_ESM.pdf]

nature research

Corresponding author(s): Diether Lambrechts

Last updated by author(s): May 18, 2021

Reporting Summary

Nature Research wishes to improve the reproducibility of the work that we publish. This form provides structure for consistency and transparency in reporting. For further information on Nature Research policies, see our Editorial Policies and the Editorial Policy Checklist.

Statistics

For all statistical analyses, confirm that the following items are present in the figure legend, table legend, main text, or Methods section.

n/a	Confirmed	
☐	☒	The exact sample size (*n*) for each experimental group/condition, given as a discrete number and unit of measurement
☐	☒	A statement on whether measurements were taken from distinct samples or whether the same sample was measured repeatedly
☐	☒	The statistical test(s) used AND whether they are one- or two-sided<br>*Only common tests should be described solely by name; describe more complex techniques in the Methods section.*
☐	☒	A description of all covariates tested
☐	☒	A description of any assumptions or corrections, such as tests of normality and adjustment for multiple comparisons
☐	☒	A full description of the statistical parameters including central tendency (e.g. means) or other basic estimates (e.g. regression coefficient) AND variation (e.g. standard deviation) or associated estimates of uncertainty (e.g. confidence intervals)
☐	☒	For null hypothesis testing, the test statistic (e.g. *F*, *t*, *r*) with confidence intervals, effect sizes, degrees of freedom and *P* value noted<br>*Give P values as exact values whenever suitable.*
☐	☒	For Bayesian analysis, information on the choice of priors and Markov chain Monte Carlo settings
☐	☒	For hierarchical and complex designs, identification of the appropriate level for tests and full reporting of outcomes
☐	☒	Estimates of effect sizes (e.g. Cohen's *d*, Pearson's *r*), indicating how they were calculated

Our web collection on statistics for biologists contains articles on many of the points above.

Software and code

Policy information about availability of computer code

Data collection	Raw sequencing reads were obtained 1) from low-coverage whole- genome sequencing on a HiSeq platform (Illumina, San Diego, CA, USA) using a V4 flow cell generating 1 × 51 bp reads, with a median read count of 10.4·106 reads per sample, and 2) from genome-wide paired-end sequencing on a NovaSeq 6000 platform (Illumina, San Diego, CA, USA), generating 2 × 151 bp reads at coverage 7.4×, 18.8× and 30.6×.
Data analysis	Raw sequencing reads were mapped to the human reference genome Hg19 using BWA v0.7.1. Duplicate and low-quality reads were removed by Picard Tools v1.11 and Samtools v0.1.18 respectively. For genome-wide Z-score, the QDNAseq package v1.0.5 was used to assign the mapped reads to nonoverlapping, fixed-sized, autosomal bins of 1000 kb along the genome, while counting the number of reads in each bin. The interface from R with package rstan v2.18.1 was used for the Bayesian hierarchical model implemented in our nucleosome score analysis. ASCAT v2.0.7 was used for segmentation and copy number estimation of the tumor samples. The pROC package (v1.17.0.1) in R was used to construct receiver operation characteristic curves and to calculate the corresponding area under the curve values. All data was processed in R version 3.1.3. GNU parallel was used for running scripts in parallel. Datasets from Snyder et al. (Cell 2016) and from Despierre et al. (Gynecol Oncol 2014) were used as reference dataset and comparative dataset, respectively.

For manuscripts utilizing custom algorithms or software that are central to the research but not yet described in published literature, software must be made available to editors and reviewers. We strongly encourage code deposition in a community repository (e.g. GitHub). See the Nature Research guidelines for submitting code & software for further information.

Data

Policy information about availability of data

All manuscripts must include a data availability statement. This statement should provide the following information, where applicable:

- Accession codes, unique identifiers, or web links for publicly available datasets
- A list of figures that have associated raw data
- A description of any restrictions on data availability

Low-coverage whole-genome sequencing data of the 271 patients and 125 healthy individuals have been deposited under restricted access in the European Genome-phenome Archive (EGA) under study no. EGAS00001005361. Requests for accessing raw sequencing reads will be reviewed by the UZLeuven-VIB data access committee. Any data shared will be released via a Data Transfer Agreement that will include the necessary conditions to guarantee protection of personal data (according to the European GDPR law).
Datasets from Snyder et al. (Cell 2016) and from Despierre et al. (Gynecol Oncol 2014) were used as reference dataset and comparative dataset, respectively.

Field-specific reporting

Please select the one below that is the best fit for your research. If you are not sure, read the appropriate sections before making your selection.

☒ Life sciences ☐ Behavioural & social sciences ☐ Ecological, evolutionary & environmental sciences

For a reference copy of the document with all sections, see nature.com/documents/nr-reporting-summary-flat.pdf

Life sciences study design

All studies must disclose on these points even when the disclosure is negative.

Sample size

Low-coverage whole genome sequencing (LC-WGS) was performed on 1) plasma samples from the discovery cohort and on 2) plasma samples and matched FFPE tumor biopsy samples from the validation cohort:
1) The discovery cohort included:
- Blood samples 125 from healthy female individuals. This group consisted of healthy donors and of patients consulting the hospital for non-ovarian related gynaecological complaints; the latter were only included after transvaginal ultrasound demonstrating two normal ovaries.
- Baseline plasma samples from 43 patients with relapsed high-grade serous ovarian cancer (HGSOC). These samples were the first batch of available baseline blood samples (n=43) obtained from patients participating in the phase 2 GANNET53 trial (NCT02012192). This trial included female patients with platinum-resistant relapsed ovarian cancer, treated with paclitaxel with or without the Hsp90-inhibitor ganetespib.
2) The validation cohort (n=271) included:
- Pre-treatment blood samples from 271 patients with an adnexal mass undergoing surgical treatment, of which 130 exhibited on pathological examination a benign adnexal mass, 41 had a borderline ovarian tumor (BOT), 92 exhibited invasive ovarian disease and 8 cases presented with adnexal metastases of a non-ovarian malignancy. Patients were consecutively enrolled in the TRANS-IOTA study (NCT01698632 and NCT02847832) after diagnosis with transvaginal ultrasound at the University Hospitals Leuven (Belgium) between June 2015 and February 2017. Exclusion criteria were presence of or active therapy for non-ovarian cancer at the moment of inclusion, presence of immune disease, treatment with immunomodulators, pregnancy, age below 18 years, surgery of the suspected mass elsewhere prior to inclusion and positive infectious serology (HIV, HepB, HepC).
- 19 matching formalin-fixed paraffin-embedded (FFPE) tumor biopsy samples from non-HGSOC plasma samples

Additionally, 3 plasma samples with high tumoral load were selected for genome-wide paired-end sequencing

There was no sample size calculation performed, because it is an exploratory study.

Data exclusions

NA

Replication

Only biological replicates were used.

Randomization

No randomization was performed.

Blinding

NA

Reporting for specific materials, systems and methods

We require information from authors about some types of materials, experimental systems and methods used in many studies. Here, indicate whether each material, system or method listed is relevant to your study. If you are not sure if a list item applies to your research, read the appropriate section before selecting a response.

Materials & experimental systems

n/a	Involved in the study
☒	☐ Antibodies
☒	☐ Eukaryotic cell lines
☒	☐ Palaeontology and archaeology
☒	☐ Animals and other organisms
☐	☒ Human research participants
☐	☒ Clinical data
☒	☐ Dual use research of concern

Methods

n/a	Involved in the study
☒	☐ ChIP-seq
☒	☐ Flow cytometry
☒	☐ MRI-based neuroimaging

Human research participants

Policy information about studies involving human research participants

Population characteristics

All study participants were female.
Discovery cohort:
- The healthy controls (negative controls) consisted of healthy donors and of patients consulting the hospital for non-ovarian related gynaecological complaints. Their median age was 52 years.
- Patients with relapsed HGSOC. Their median age was 62 years. These patients had platinum-resistant relapsed ovarian cancer and were treated with paclitaxel with or without the Hsp90-inhibitor ganetespib.

Validation cohort:
Patients with an adnexal mass, undergoing surgical treatment. Patients were consecutively enrolled in the TRANS-IOTA study (NCT01698632 and NCT02847832) after diagnosis with transvaginal ultrasound at the University Hospitals Leuven. Age, BMI, final histology and FIGO stage were collected and summarized in Table 1. Exclusion criteria were presence of or active therapy for non-ovarian cancer at the moment of inclusion, presence of immune disease, treatment with immunomodulators, pregnancy, age below 18 years, surgery of the suspected mass elsewhere prior to inclusion and positive infectious serology (HIV, HepB, HepC).

Recruitment

In the discovery cohort, the healthy control group consisted of healthy donors and of patients consulting the hospital for non-ovarian related complaints with two normal ovaries after transvaginal ultrasound, and the patient group consisted of relapsed HGSOC patients participating in the phase 2 GANNET53 trial (NCT02012192).
In the validation cohort, patients with an adnexal mass, undergoing surgical treatment and after diagnosis with transvaginal ultrasound at the University Hospitals Leuven were included in this study and enrolled in the TRANS-IOTA study (NCT01698632 and NCT02847832). Exclusion criteria were presence of or active therapy for non-ovarian cancer at the moment of inclusion, presence of immune disease, treatment with immunomodulators, pregnancy, age below 18 years, surgery of the suspected mass elsewhere prior to inclusion and positive infectious serology (HIV, HepB, HepC).

Ethics oversight

The Ethics Committee Research UZ/KU Leuven approved this study (study numbers S64035 and S64205 for the controls, and S51375 and S59207 for the patients with an adnexal mass). All study participants provided written informed consent. The study was conducted according to EU legislation regarding ethical regulations and was registered online (NCT02012192, NCT01698632 and NCT02847832).

Note that full information on the approval of the study protocol must also be provided in the manuscript.

Clinical data

Policy information about clinical studies
All manuscripts should comply with the ICMJE guidelines for publication of clinical research and a completed CONSORT checklist must be included with all submissions.

Clinical trial registration

ClinicalTrials.gov Identifier: NCT02012192, NCT01698632 and NCT02847832

Study protocol

NCT02012192: GANNET53: Ganetespib in Metastatic, p53-mutant, Platinum-resistant Ovarian Cancer: https://clinicaltrials.gov/ct2/show/NCT02012192
NCT01698632: International Ovarian Tumour Analysis (IOTA) Phase 5 (IOTA-5): https://clinicaltrials.gov/ct2/show/NCT01698632
NCT02847832: Prospective Validation and Comparison of Different Ultrasound Methods for Discrimination Between Benign and Malignant Ovarian/Tubal Masses Prior to Surgery (IOTA7): https://clinicaltrials.gov/ct2/show/NCT02847832.
This study investigated the secondary endpoint of the clinical trials IOTA-5 and IOTA-7.

Data collection

Blood samples and FFPE tumor tissue samples from the validation cohort and control samples have been obtained at the University Hospitals Leuven (UZLeuven), Leuven, Belgium. Blood samples from the patients in the discovery cohort were collected via the GANNET53 trial. (Cell-free) DNA extraction has been performed at the Department of Oncology (KULeuven) and the Laboratory of Translational Genetics (VIB and KULeuven, Leuven, Belgium), which both are on the same university campus as the hospital (Gasthuisberg). All subsequent wet-lab experiments were performed at the Laboratory for Translational Genetics. High-throughput sequencing was done on an Illumina HiSeq and NovaSeq housed by the Genomics Core at KULeuven. Data analysis was performed using computational resources and (secured and password-protected) services provided by the VSC (Flemish Supercomputer Center, Belgium at KULeuven).

Outcomes

To assess whether low coverage whole genome sequencing (LC-WGS) can be used to detect invasive ovarian tumors by assessing the

Outcomes

nucleosome footprints in plasma-derived cell-free DNA, and to assess whether combining chromosomal instability and nucleosome footprinting (both reads out from LC-WGS) in cell-free DNA is more reliable in detecting invasive ovarian tumors in women with an adnexal mass than either method alone.
